# Development of a Benchmarking Tool for Dairy Herd Management Using Routinely Collected Herd Records

**DOI:** 10.3390/ani10091689

**Published:** 2020-09-18

**Authors:** Daniel Warner, Elsa Vasseur, Marianne Villettaz Robichaud, Steve Adam, Doris Pellerin, Daniel M. Lefebvre, René Lacroix

**Affiliations:** 1 Lactanet, Canadian Network for Dairy Excellence, Sainte-Anne-de-Bellevue, QC H9X 3R4, Canada; dwarner@lactanet.ca (D.W.); sadam@lactanet.ca (S.A.); dlefebvre@lactanet.ca (D.M.L.); 2 Department of Animal Science, McGill University, Sainte-Anne-de-Bellevue, QC H9X 3V9, Canada; elsa.vasseur@mcgill.ca; 3 Département des Sciences Cliniques, Université de Montréal, Saint-Hyacinthe, QC J2S 2M2, Canada; marianne.villettaz.robichaud@umontreal.ca; 4 Département des Sciences Animales, Université Laval, Québec, QC G1V 0A6, Canada; Doris.pellerin@fsaa.ulaval.ca

**Keywords:** dairy herd improvement, composite index, animal welfare, animal health, profitability

## Abstract

**Simple Summary:**

Continuous monitoring of the herd status is important but routine assessments on farm can be challenging. In this study, a remote herd assessment tool was developed to help producers and advisors detect herd management issues related to herd welfare and health. This tool was based on pre-recorded indicators from routinely collected on-farm records. Indicators were aggregated to a composite index to benchmark the overall herd status, with a large composite index indicating an overall high herd status and a small composite index indicating an overall low herd status. Robustness of the composite index was evaluated and indicated little fluctuation for herds with a low or high status. These results suggest that herds in need of support could be prioritized and effectively monitored over time, limiting the need for time-consuming farm visits. The benchmarking tool allows evaluating herds relative to their peers, highlights areas with opportunities to improve, may be further suitable for certification systems, and may be applied to studies to benchmark multidimensional aspects of livestock farming such as environmental and socio-economic studies.

**Abstract:**

Continuous assessment of the herd status is important in order to monitor and adjust to changes in the welfare and health status but can be time consuming and expensive. In this study, herd status indicators from routinely collected dairy herd improvement (DHI) records were used to develop a remote herd assessment tool with the aim to help producers and advisors benchmark the herd status and identify herd management issues affecting welfare and health. Thirteen DHI indicators were selected from an initial set of 72 potential indicators collected on 4324 dairy herds in Eastern Canada. Data were normalized to percentile ranks and aggregated to a composite herd status index (HSI) with equal weights among indicators. Robustness analyses indicated little fluctuation for herds with a small HSI (low status) or large HSI (high status), suggesting that herds in need of support could be prioritized and effectively monitored over time, limiting the need for time-consuming farm visits. This tool allows evaluating herds relative to their peers through the composite index and highlighting specific areas with opportunities for improvements through the individual indicators. This procedure could be applied to similar multidimensional livestock farming issues, such as environmental and socio-economic studies.

## 1. Introduction

Assessing the overall status of a dairy herd can be challenging as it affects various important aspects of dairy farming such as welfare, health, reproduction and production performance, and profitability. Continuous assessments on farm are necessary to monitor the herd status but collecting animal-based measures is time consuming and expensive [1,2]. Therefore, to monitor a large number of herds on a regular basis, a remote herd assessment tool would be a practical option. Ideally, such an assessment tool would be based on herd data that are routinely collected. Particularly with regard to herd welfare, associations of routine herd data with one or more welfare indicators were extensively reviewed elsewhere [3]. Previous studies conducted in Europe validated the use of dairy herd improvement (DHI) data to assess herd welfare [4,5,6]. Such DHI records typically consist of routinely collected herd records relating to identification and registration, housing, productivity, milk quality, fertility, health, longevity, and profitability for lactating cows and youngstock.

Previous initiatives have further looked at a multicriteria evaluation approach by aggregating potential indicators to evaluate complex issues such as on-farm herd welfare [4,5,7,8], and sustainable farming such as the Farm Sustainability Indicators assessment [9], the Environmental Performance Index [10], and the Agro-System Performance Index [11]. These studies typically intend to evaluate key factors and highlight individual strengths and weaknesses to offer opportunities for improvement through benchmarking, i.e., provide an overall herd assessment compared to peers. 

The objective of this study was to develop a tool to assess, monitor, and benchmark the overall herd management, status covering key aspects of welfare and health such as longevity, reproduction, nutrition, production, young stock management, and profitability. We propose an approach based on a composite herd status index (HSI) composed of individual indicators linked to key aspects of herd welfare and health. As DHI records can differ among various countries, this paper does not aim at identifying an absolute set of indicators for defining an acceptable or good herd status, but to propose a dynamic approach that can be generalized and applied to other farm records. 

## 2. Materials and Methods 

### 2.1. Selection of Indicators

Records for 4324 dairy herds in Eastern Canada (the Atlantic Provinces and Quebec) from September 2016 to September 2017 were extracted from the DHI database at Lactanet (Sainte-Anne-de-Bellevue, QC, Canada). All data were expressed at the herd level as an average over the study period. Herd records with less than three available test dates over the study period and herds with less than 10 cows were removed. Cows were housed in tie stalls (83%) and free stalls (17%), the latter equipped with either a milking parlour (10%) or an automatic milking system (7%). The predominant breed was Holstein Friesian (91%), followed by Ayrshire and Jersey (4% each). Average herd size was 60 ± 28 (mean ± SD) cows for tie stalls, and 111 ± 83 cows for free stalls. Average milk production per year was 9364 ± 1504 kg for tie stalls, and 9717 ± 1569 kg for free stalls. 

Initially, 72 pre-recorded variables assembled in a DHI database were assessed to be part of the benchmarking tool (Table 1). Data included information relating to identification and registration, housing, productivity, milk quality, fertility, longevity, and profitability. Potential correlation among these indicators was evaluated through bivariate correlation analysis. If pairs of variables were correlated to each other, only one was selected based on the proportion of missing values, reliability of measurement, and ease of data collection. Regarding the latter two selection criteria, a group of 10 senior advisors from research and DHI stations across Eastern Canada with their main expertise in either animal welfare, health, nutrition, economics, data analytics or farm management was consulted. In addition, potentially relevant indicators that could not be addressed by the farmer in a reasonable period or that were simply the result of the producer’s choice (e.g., type of housing and organic status) were excluded, as the objective was to allow producers to take timely actions to improve their herd status. The final set of 13 indicators covered key aspects of herd welfare and health such as longevity, reproduction, nutrition, production, young stock management, and profitability (Table 2). 

### 2.2. Aggregation to a Composite Index

A composite index was calculated for each herd using the 13 individual indicators described above. As distribution among indicators varied, the individual indicators were normalized by transforming values to percentile ranks. Indicators for which a lower prevalence is desirable (e.g., mortality rate) were normalized by transforming values to the reciprocal of the percentile rank. 

This approach prevented extreme values from distorting the HSI and allowed for the comparison of indicators expressed in different units within and among herds. Moreover, transformation to percentile ranks enabled assessing the performance of a herd relative to its peers. Other common methods of transformation such as transforming to standard scores and rescaling to range are discussed in this study as modifications in the computation of the HSI may affect the herd ranking and interpretation of the HSI. 

Transformed values of the 13 individual indicators were aggregated to a unique HSI based on a linear additive aggregation with equal weights for each indicator:

HSI = (∑ IND_rank_)/*N* (1)

where IND_rank_ denotes the percentile ranks for each of the 13 individual indicators, and *N* denotes the number of individual indicators. 

**Figure 1:**
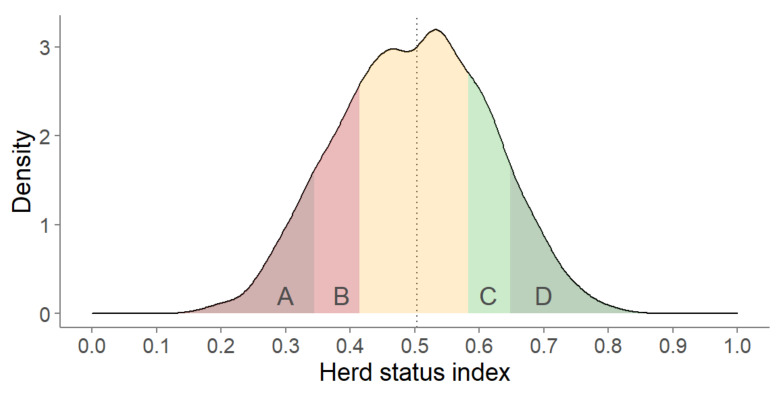
Distribution of the composite herd status index (HSI) across 4321 dairy herds for herds in the bottom 10% (**A**), bottom 25% (**B**), median (vertical line), top 25% (**C**), and top 10% (**D**), with a small HSI value suggesting a low overall herd status.

The HSI was then transformed to percentile ranks, resulting in a normal distribution of HSI across the population (Figure 1). Herds with missing data for more than three indicators were removed, resulting in a study population of 4321 herds. All data handling and calculation steps were conducted in R (version 3.5.0; R Foundation for Statistical Computing, Vienna, Austria). 

### 2.3. Validation of the Composite Index

Multicollinearity analysis in combination with bivariate correlation analysis and a principal component analysis were conducted in R 3.5.0 to assess a potential collinearity among the final set of individual indicators. A strong interrelation among indicators may distort the HSI, as those indicators will have a stronger weight on the composite index. Associations among the HSI and the individual indicators were estimated through Kendall rank correlations to handle the asymmetrical distribution and extreme values for some indicators in the dataset. Size effects of individual indicators on the HSI were evaluated based on differences between the means of the top 25% and bottom 25% herds using Cohen’s d parameter and proposed thresholds [13,14]. 

A robustness analysis of the composite index was conducted to verify whether herds might be assigned different ranks depending on the underlying indexing method and, thus, to assess whether future modifications to the composite index might render it unstable (i.e., if the herd ranking is affected). Robustness was assessed as recommended by the Organisation for Economic Co-operation and Development (OECD) [15] by analysing how uncertainty inherent to the input factors (the individual indicators) affects the composite index. In line with the OECD recommendations [15], we looked at three types of uncertainty: (i) uncertainty due to the inclusion or exclusion of specific indicators; (ii) uncertainty due to an alternative data normalization of the individual indicators; (iii) and uncertainty due to an alternative aggregation of the individual indicators. The various indexing methods in points (i) through (iii) are described in more detail in Appendix A. Robustness of the composite index was evaluated by calculating the mean and SD of HSI for each herd among all possible variations in the indexing method. 

In addition, robustness of the composite index over time was assessed by comparing the median absolute difference of the original HSI (4321 herds from September 2016 to September 2017) with the HSI generated from indicators covering the following period (4003 herds from September 2017 to September 2018). 

## 3. Results and Discussion

### 3.1. Composite Herd Status Index

A multidimensional approach was used to calculate a composite index based on 13 indicators selected to cover various aspects of herd management affecting herd welfare and health, extracted from DHI records for all registered herds. These indicators were not strongly correlated to each other as suggested by a tolerance test of minimum 0.99 and a variance inflation factor of maximum 5.4 [16]). A principal component analysis revealed that six principal components were needed to account for at least 66% of the variance in the dataset. These results suggest that the individual indicators were not strongly correlated with each other. 

**Figure 2:**
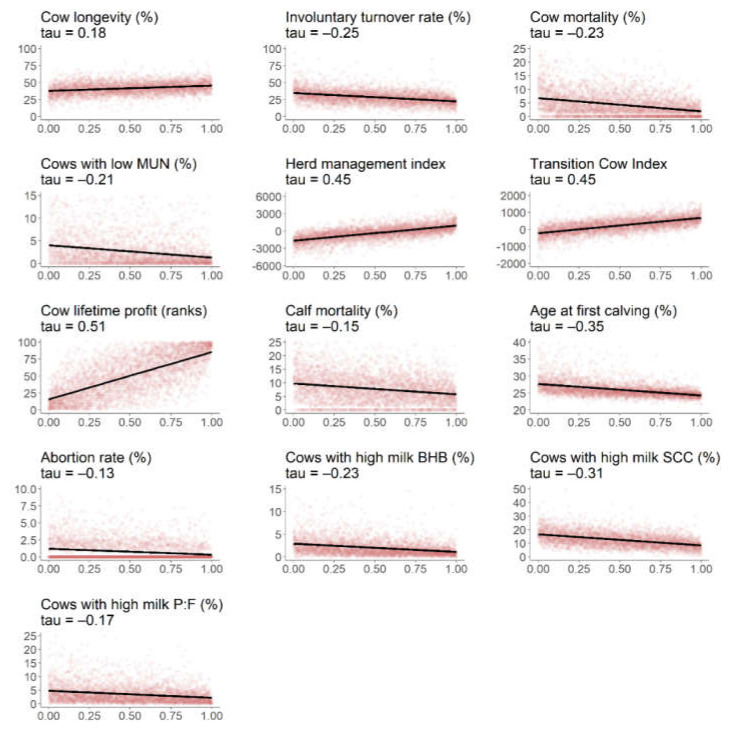
Linear relationship (*p* < 0.001) between the composite herd status index (*x* axis) and individual indicators (*y* axis) based on Kendall’s tau rank correlation coefficient across 4321 dairy herds registered in the dairy herd improvement database (abbreviations: MUN = milk urea nitrogen; BHB = ß-hydroxybutyrate; SCC = somatic cell count; P:F = protein-to-fat ratio).

The strength of association between the individual indicators and HSI differed (Figure 2). Associations with the HSI were strongest for production-related indicators such as cow lifetime profit, herd management index, and transition cow index (Kendall’s tau ≥ 0.45). The HSI was most weakly associated with calf mortality and abortion rate, presumably due their low prevalence and skewed distribution. Additional information, if available, on calf mortality at later calf development stages [4] might strengthen the association with HSI. Moreover, converting absolute values to a relative change (e.g., the relative change in calf mortality over consecutive years) might circumvent the issue of low prevalence and heavily skewed distributions. Although the aim of the present study was not to assess direct associations with welfare and health, previous studies reviewed elsewhere [3] have shown that routine herd data relating to milk yield, culling, and reproduction were well associated with important aspects of herd welfare such as lameness, body lesions, and cleanliness. 

### 3.2. Robustness of the Composite Index

The tool proposed in this study may likely undergo modifications, mainly with regard to the choice of indicators, as DHI records are continuously updated and grow in terms of volume and diversity. Future modifications in the composite index computation might render the HSI unstable [15]. Therefore, the robustness of the composite index and potential sources of uncertainty in the development of the composite index were assessed to verify whether herds might be assigned different ranks depending on the underlying method for HSI calculation (see Appendix A for a detailed description of the various indexing methods). 

**Figure A1:**
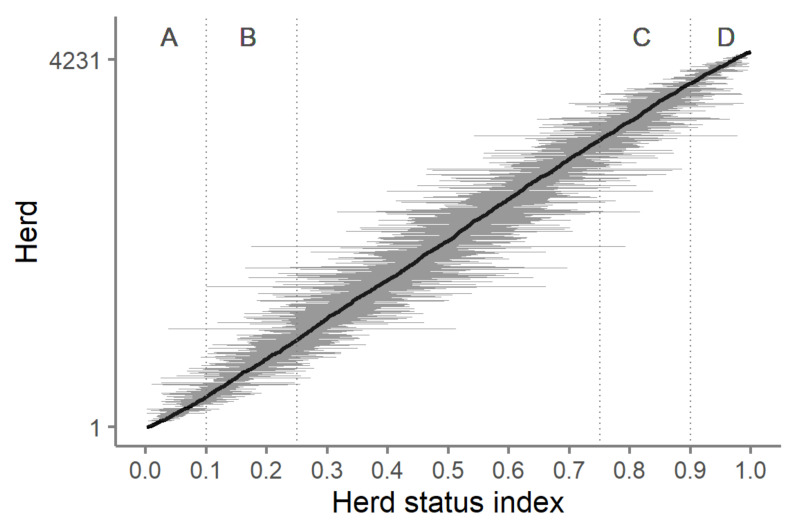
Robustness of the composite herd status index (HSI) with the herd-specific mean (diagonally aligned dots arranged by ascending HSI) and the respective standard deviation (horizontal lines) for herds in the bottom 10% (**A**), bottom 25% (**B**), top 25% (**C**) and top 10% (**D**) across 4321 dairy herds.

Some uncertainty related to the indexing method was observed (Figure A1; see Appendix A); however, the HSI fluctuated less for low-ranking herds (<p10; SD of 0.066) and for high-ranking herds (>p90; SD of 0.062) compared to average herds (SD of 0.162 for herds ranking between p25 and p75). These results suggest that the proposed approach might be useful to monitor herds that rank at the top and bottom in term of herd status. Herds in need of support may be prioritized, limiting the need for time-consuming farm visits.

In-depth analysis revealed that the variation in HSI originated at an equal proportion from the three indexing methods texted in the present study (i.e., data selection, data normalization, and data aggregation), with a coefficient of variation (CV) of 0.18‒0.19. Overall, herds with the smallest (<p10) variation in HSI showed considerably less fluctuation across the 13 individual indicators (mean CV of 0.29 across all indicators) as compared to herds with the largest (>p90) variation in HSI (mean CV of 0.92). These results suggest that stability of HSI was mainly influenced by the presence of extreme values for the individual indicators. Careful removal of extreme values might improve the overall robustness of the HSI to some extent. The concerned herds can be identified from Figure A1 (see Appendix A) and can be extracted for further in-depth studies. 

A comparison of the HSI computed for two subsequent years (2016–2017 versus 2017–2018 period) revealed that the ranking of herds fluctuated to some extent between the 2 years. The median absolute difference in HSI across all herds between the 2 years was 0.11; however, the low-ranking (<p10) and high-ranking herds (>p90) in the first year fluctuated considerably less (median absolute difference in HSI of 0.05, respectively) than the average herds (median absolute difference in HSI of 0.14 for herds ranking between p25 and p75). 

These results suggest that herds with a low and high status are relatively stable over time. Although certain indicators may fluctuate to some extent, large variations are not to be expected for these herds from one year to another. Herds with a high status are evidently classified as such because of their high performance across all indicators. Herds with a low status will however require extensive changes in overall herd management. 

These findings suggest that herds with a low welfare and health herd status can be targeted using the proposed DHI variables, and advisory service effectively allocated. Comparison with other studies is not possible at this stage as, to our knowledge, similar sensitivity tests have not been reported previously. It should however be noted that these fluctuations could reflect to some extent potential changes in management or housing made by farmers over the 2 years that are not accounted for in the DHI records. 

### 3.3. Composite Index Approach

Aggregating scores into an overall assessment offers the opportunity to provide a broad assessment of herd status. However, the usefulness of such an approach depends heavily on the integration of on-farm records and standardization of measurements. On-going collaborative research efforts into standardizing welfare indicators and scoring methods such as those for lameness, body injuries, or cleanliness [17] are expected to facilitate their integration and allow comparison between countries.

Aggregation of individual indicators may mask the relative contribution of weaker yet potentially relevant indicators on the overall herd status [18]. In the present study, indicators were equally weighed to prevent specific indicators compensating for others (e.g., a high production performance cannot compensate for lesser reproduction performance). If specific aspects of herd welfare and health are of interest, the indicators may be individually weighed by attributing a relative importance to indicators, and knockout thresholds can be assigned that indicators must meet to be included in the aggregation. With this latter approach, if a herd fails to meet a minimum threshold, it will score lower or be excluded altogether, independent of how well the herd performs on other indicators. This approach might be useful for certification systems (e.g., by promoting management strategies leading to improved welfare for house-stalled herds). 

**Figure 3:**
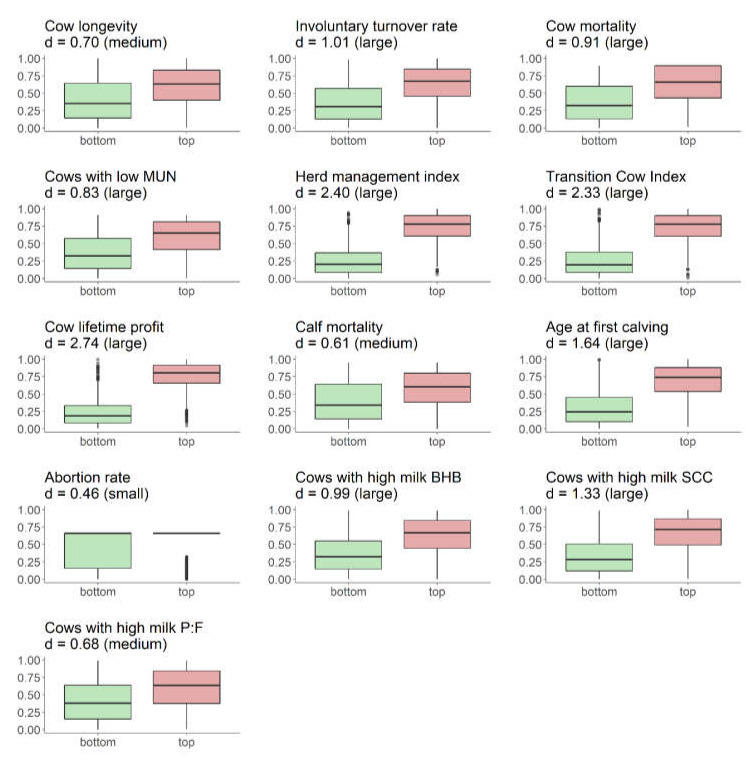
Distribution of individual indicators (y axis) expressed as percentile ranks for herds in the bottom 25% (*n* = 1081) and top 25% (*n* = 1080) across 4321 dairy herds (differences between means based on Cohen’s d effect size statistic as small, medium and large effect; horizontal line: mean percentile rank of population; boxplots: box with interquartile range and median, whiskers with minima and maxima, and dots with extreme values; MUN = milk urea nitrogen; BHB = ß-hydroxybutyrate; SCC = somatic cell count; P:F = protein-to-fat ratio).

Our results suggest that herds with a low and high status can be well distinguished, with most indicators having a distinct effect on the observed difference between herds in the top 25% and bottom 25% (Figure 3). The effect size was largest for production-related indicators (cow lifetime profit, herd management index, and transition cow index), in line with results from Figure 2. A medium effect was observed for cow longevity, calf mortality, and percentage of cows with a high milk protein-to-fat ratio, whereas only a small effect was observed for abortion rate, which is consistent with their overall low prevalence observed in the present study. 

### 3.4. Practical Implications and Limitations

The multicriteria evaluation approach described in the present study can be used routinely to assess the herd status on a large number of herds and can be thus an interesting tool to improve continuously herd welfare and the herd health status on large scale. By ranking and comparing herds, the individual herd status can be continuously improved, even if the overall herd status or specific indicators such as the overall herd welfare level gradually increases [15]. 

As this approach does not allow assessing issues at the animal level such as individual animal welfare or health, the individual cow status might not be necessarily adequate despite an overall inconspicuous status at the herd level. Moreover, a comprehensive assessment of individual key aspects is not possible with this approach; for instance, for a comprehensive welfare assessment, further indicators that are typically not routinely measured and assembled in current DHI databases such as natural living and affective states of the animals [19] may have to be considered. Assessing the overall herd level status can be nonetheless useful in targeting low- or high-performing herds and allocating advisory services effectively [4,5]. 

The selected set of indicators originated from pre-collected routine data and is therefore not a comprehensive list. However, additional indicators may be integrated in the future such as routine measurements of the affective states of animals using kinematics [20], and activity- and health-related indicators from sensor data or from advanced predictive analytical methods using machine learning [21,22]. It should be noted that this paper did not aim at identifying an absolute set of indicators for defining an acceptable or good herd status. The inherent flexibility of the approach described in the present study allows for different applications and for the inclusion of different farm records depending on the availability of data and the objective of this study. For instance, for a study mainly focussed on farm profitability, a larger number of profitability indicators should be considered if available or higher weights could be assigned for profitability indicators as compared to other indicators less linked to herd profitability. Nonetheless, the indicators described in this study need to be confirmed in future studies. In addition, give the high number of tie-stall herds in the present study (83%), the indicators should be confirmed for free-stall or pasture-based systems. 

Although a monitoring instrument based on routinely collected DHI records might be considered a valid alternative to a standardized herd check [23], concerns about the completeness and reliability of the collected data should be considered and might limit their use. Typically, DHI records are not available for all herds registered in a national database, are not collected on the same frequent basis throughout all herds, and producers may stop collecting certain data. The reliability and consistency may vary among individual DHI records or among DHI agencies in the various countries and should be verified. Nonetheless, DHI records were found useful to assess multidimensional and dynamic herd issues such as herd welfare [4,5,6].

Our results suggest that the method is repeatable, but care should be taken for herds with extreme values for some indicators, as those herds might be more prone to an unstable composite index. Nonetheless, this effect on the composite index can serve as an incentive to improve quality of data collection and raise awareness among stakeholders on the importance of accurate data collection. From an advisory service point of view, the evaluation tool can be explained relatively easily to stakeholders as it focuses on a set of important herd welfare and health indicators. It can encourage participation as it allows producers benchmarking the herd performance relative to their peers and allows developing improvement strategies specific to the individual herd [24]. This approach might be also used in certification systems based on several different welfare and health aspects. 

## 4. Conclusions

The remote assessment tool proposed in the present study allows benchmarking the herd management status in dairy herds kept under stall conditions based on a predetermined set of routinely collected indicators. The herd status can be continuously assessed and monitored without the need for time-consuming and expensive individual farm visits. This tool allows highlighting individual herd strengths and weaknesses related to 13 potential indicators, covering key aspects of herd welfare and health such as longevity, reproduction, nutrition, production, young stock management, and profitability. The tool further provides stakeholders with an overall herd evaluation compared to their peers. Individual herds of interest can be identified, such as low-performing herds or herds experiencing sudden and large changes to help producers adopt tailored strategies. Likewise, high-performing herds can be identified to evaluate their strengths and management strategies in more detail. The multicriteria evaluation approach described in the present study might be used with other national herd databases and might be generalized for other benchmark studies based on a multidimensional approach, such as environmental and socio-economical aspects of livestock farming. 

## Figures and Tables

**Table 1:** Indicators from pre-collected data registered in the dairy herd improvement database with superscripts explaining the decision rules applied for exclusion.

Category	Indicator
Identification and registration	Cohort (birth year) ^1^, birth date ^1^, herd size ^1^, region ^1^, type of housing ^1^, milking system ^1^, certified organic ^1^, breed ^1^
Longevity	Number primiparous ^1^, number heifers ^1^, number lactating cows ^1^, persistency lactation ^1^, age at first calving, calf mortality 0–24 h, calf mortality >24 h ^2^, heifer mortality ^2^, heifers dead ^2^, cow mortality, cow longevity, turnover rate ^1^, voluntary turnover ^1^, involuntary replacement for cows, total involuntary replacement for heifers ^2^, number cows left ^1^, number cows left at 60 DIM ^1^, cows sold ^1^, cows dead ^1^, cows left for reproduction issues ^2^, cows left for mastitis ^2^, cows left for feet problems ^2^, cows left for unknown reasons^1^
Nutrition, production and profitability	Days in milk ^1^, milk yield ^1^, milk fat yield ^1^, milk protein yield ^1^, milk lactose yield ^1^, body weight for lactating cows ^3^, fat ^1^, protein^1^, lactose^1^, milk urea nitrogen (MUN) ^1^, somatic cell count (SCC) ^1^, somatic cell score^1^, beta-hydroxybutyrate (BHB) ^1^, freezing point ^1^, cows with low MUN (<5), cows with high MUN (>18) ^1^, cows with high milk protein-to-fat ratio (<1.1),genetic potential ^1^, genetic index for milk ^1^, genetic index for milk fat ^1^, genetic index for milk protein ^1^, herd management index ^4^, herd management index for milk ^1^, herd management index for milk fat ^1^, herd management index for milk protein ^1^, transition cow index ^5^, cow lifetime profit rank ^6^
Young stock, reproduction	Calving date ^1^, daily gain 0–15 months ^3^, daily gain 15–24 months ^3^, body condition score (BCS) ^3^, cows with low and high BCS ^3^, age at first calving, average calving interval ^1^, pregnancy rate ^1^, days dry ^1^, days to breeding ^1^, number of breeding service per cow ^1^, cows with high BHB (>0.2), cows with high SCC (>400,000), abortion rate

^1^ Not selected due to collinearity with retained indicators, or because changes are not feasible within a reasonable period or due to the producer’s choice (e.g., type of housing). ^2^ Not selected due to concerns about data quality (i.e., data not uniformly recorded across herds). ^3^ Not selected due to small proportion of herds with available records. ^4^ Indicator of the optimal use of the genetic potential based on standardized milk (0.2594 × kg milk + 12.1975 × kg milk fat + 7.707 × kg milk protein). ^5^ Measure of the effectiveness of a herd’s transition cow program (TCI; AgSource Cooperative Services, Verona, WI, USA; U.S. Patent #7866691, Wisconsin Alumni Research Foundation; Nordlund 2006 [12]). ^6^ Indicator of estimated profitability of animals in the herd (milk revenues minus rearing, maintenance and production-related expenses).

**Table 2:** Final set of indicators with the respective herd prevalence for the study population of 4324 dairy herds.

Indicators	Herd Prevalence				
	Mean	p1	Median	p99	N
Longevity					
Cow longevity, ^1^ %	41.4	19.2	41.4	63.9	4317
Involuntary replacement rate, ^2^ %	28.4	8.2	27.7	56.8	4315
Cow mortality, %	4.4	0.0	3.4	18.8	4317
Nutrition, production and profitability					
Cows with low MUN, ^3^ %	3.6	0.0	1.6	27.7	3227
Cows with high milk P:F ^4^ %	3.6	0.0	2.6	17.0	4294
Herd management index ^5^	−362	−3597	−321	2459	4207
Transition cow index ^6^	225	−838	239	1191	4318
Cow lifetime profit rank ^7^	50.4	2.0	50.5	99.0	4210
Young stock and reproduction					
Calf mortality, ^8^ %	8.1	0.0	7.4	27.4	4320
Age at first calving, months	26.2	22.9	25.5	34.9	4321
Abortion rate, %	0.8	0.0	0.0	5.9	4321
Cows with high BHB, ^9^ %	2.1	0.0	1.6	9.5	4294
Cows with high SCC, ^10^ %	12.6	2.9	12.0	27.7	4295

^1^ Percent cows at lactation 3 or more. ^2^ Total replacement rate minus percent cows sold for production. ^3^ MUN: milk urea nitrogen; indicator of lack of dietary protein availability in the rumen (not allowing for maximum milk production); low milk urea nitrogen: <5 mg/dL of milk. ^4^ P:F ratio: protein-to-fat ratio; indicator of subacute ruminal acidosis; high milk P:F ratio: >ratio of 1.1. ^5^ Indicator of the optimal use of the genetic potential based on standardized milk (0.2594 × kg milk + 12.1975 × kg milk fat + 7.707 × kg milk protein). ^6^ Measure of the effectiveness of a herd’s transition cow program (TCI; AgSource Cooperative Services, Verona, WI, USA; U.S. Patent #7866691, Wisconsin Alumni Research Foundation; Nordlund, 2006 [12]). ^7^ Indicator of estimated profitability of animals in the herd (milk revenues minus rearing, maintenance and production-related expenses). ^8^ Calves at 0–24 h. ^9^ BHB: ß-hydroxy butyrate; measure of risk for hyperketonemia; high milk BHB: >0.2 mmol/L of milk. ^10^ SCC: somatic cell counts; indicator of mastitis; high milk SCC: >400,000 SCC/mL of milk.

## Appendix A

### Indexing Methods for Evaluating the Robustness of the Composite Index

The effect of data exclusion or inclusion (i) was tested by randomly excluding a variable among the 13 indicators. Next, an entire set of variables associated with specific features—here, all performance-related parameters were arbitrarily chosen—was excluded to simulate possible future modifications in the composite index when several new potential indicators might become available (e.g., health-related indicators from sensor data).

The effect of data normalization (ii) was tested by evaluating common normalization methods, such as transformation to standard scores, which converts indicators to a common scale with a mean of zero and SD of 1, and rescaling to range, which widens the range of indicators within a small interval. Both alternative methods may distort the composite index to some extent with the presence of extreme values.

The effect of the aggregation method (iii) was tested by comparing the linear additive aggregation (i.e., the original method described in Equation (1)) to a geometric aggregation (i.e., based on the product of all weighted indicators). Whereas a linear additive aggregation implies full compensability (i.e., poor performance in some indicators can be compensated by sufficiently high values of other indicators), a geometric aggregation is a less compensatory approach.
